# Supplementary material for: Production of an Oncolytic Adeno-Associated Virus Containing the Pro-Apoptotic TRAIL Gene Can Be Improved by shRNA Interference
Source: Int J Mol Sci. 2025 Jan 10;26(2):567. doi: 10.3390/ijms26020567 (PMC11766350; doi:10.3390/ijms26020567)
Supplement: Supplementary file 1 [file ijms-26-00567-s001.zip › ijms-3313497-supplementary.pdf]

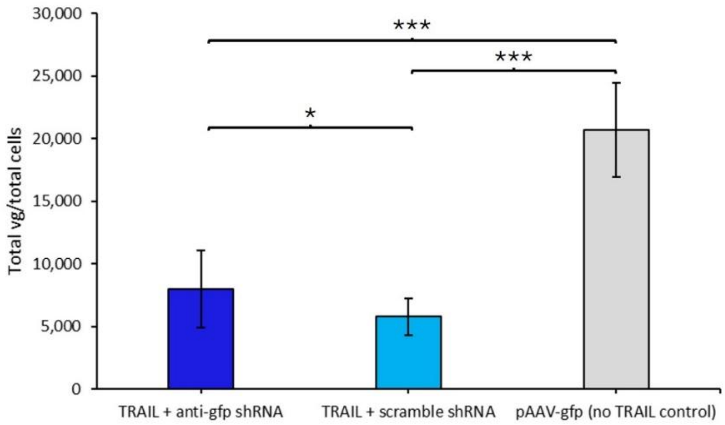

Figure S1. Total viral genomes (vg) divided by total live cells (HEK293F), 72 hrs post triple-transfection with pRep/Cap, pAdDelta and pAAV-*gfp-TRAIL* + anti-*gfp* shRNA OR pAAV-*gfp-TRAIL* + scramble shRNA OR pAAV-*gfp* (no *TRAIL* control). Each bar represents 6 biological repeats (= 6 separate transfections) for the *TRAIL* samples. pAAV-*gfp* (no *TRAIL* control) bar represents 4 biological repeats. Error bars represent +/-SD, * = $p < 0.05$, *** = $p < 0.001$ (*t*-test).

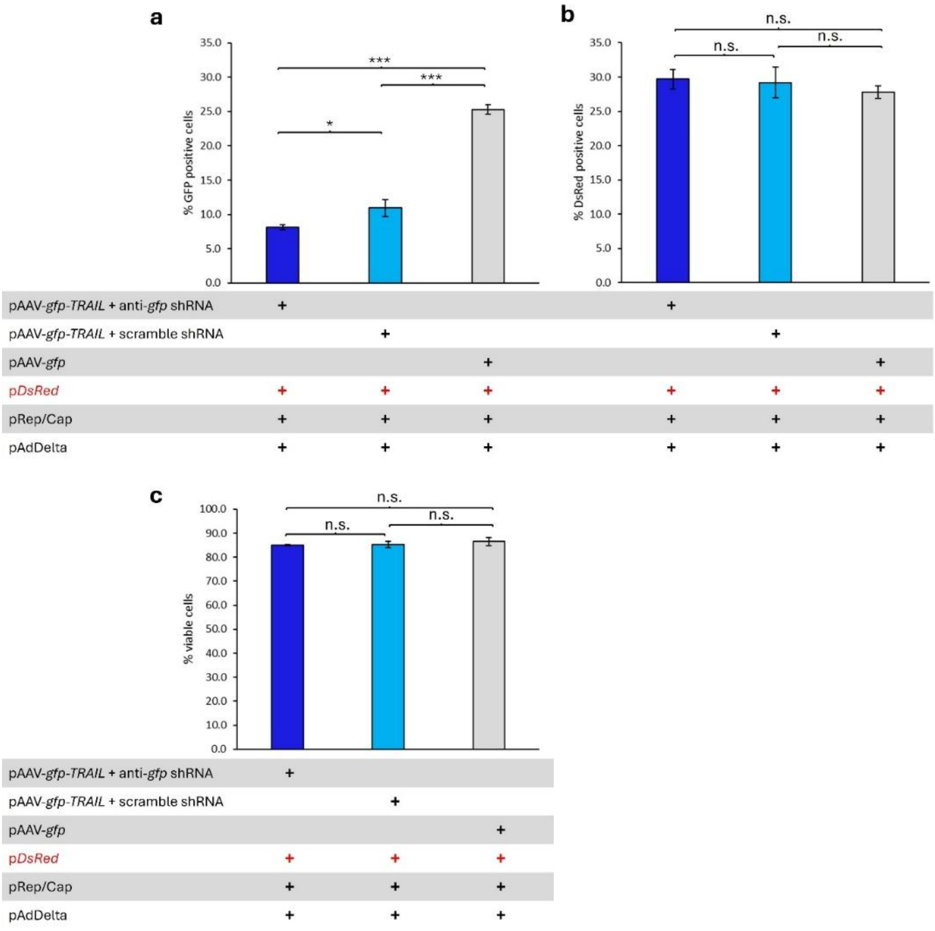

Figure S2. HEK293F cells, 72 hrs post quadruple-transfection with p*DsRed*, pRep/Cap, pAdDelta and pAAV-*gfp-TRAIL* + anti-*gfp* shRNA, or pAAV-*gfp-TRAIL* + scramble shRNA, or pAAV-*gfp*. Cells were fixed in formaldehyde, then examined by FACS Accuri C6. **a:** % GFP positive cells. **b**: % DsRed positive cells. **c:** % viable cells. Bars represent 3 separate transfections. Error bars represent +/-SD, * = $p < 0.05$, *** = $p < 0.001$ (*t*-test). n.s., not significant, *t*-test.
